# Palmoplantar Psoriasis Quality of Life Index into Brazilian Portuguese (PPQLI-BRA) ‒ Translation, cultural adaptation, and validation^؆^

**DOI:** 10.1016/j.abd.2026.501399

**Published:** 2026-06-25

**Authors:** Paula Hitomi Sakiyama, Caio Cesar Silva de Castro, Helena Zenedin Marchioro, Hélio Amante Miot

**Affiliations:** a Service of Dermatology, Universidade Estadual do Oeste do Paraná, Cascavel, PR, Brazil; b School of Medicine and Life Sciences, Pontifícia Universidade Católica do Paraná, Curitiba, PR, Brazil; c Hospital de Dermatologia Sanitária do Paraná, Piraquara, PR, Brazil; d Service of Dermatology, Hospital Santa Casa de Curitiba, Curitiba, PR, Brazil; e Department of Infectology, Dermatology, Imaging Diagnosis and Radiotherapy, Faculty of Medicine, Universidade Estadual Paulista, Botucatu, SP, Brazil

Dear Editor,

Psoriasis is a chronic, immune-mediated systemic inflammatory disease with a genetic background that affects both sexes, and with an estimated prevalence of 1.1%‒1.5% among Brazilian adults.^1^ Compared with the general population, these patients experience higher rates of anxiety, depression, and suicidal behavior.^2^ The quality-of-life (QoL) impairment is particularly severe in psoriasis on palms and soles, affecting up to 40% of patients.^3^, ^4^, ^5^, ^6^

Palmoplantar psoriasis (PP) manifests as pustular and non-pustular forms that occasionally overlap. Its chronic, treatment-resistant course causes considerable pain, loss of manual dexterity, difficulty walking, and functional disability, contributing to psychosocial distress. Recognizing this burden, the International Psoriasis Council has designated PP as a key research priority.^7^ Despite the clinical relevance of PP, no psoriasis-specific quality-of-life instrument evaluates the unique impact of palmoplantar involvement, nor does a dedicated tool currently exist for its assessment.

In 2009, Farley et al. developed a patient-recorded specific instrument to assess the QoL impact of PP, the palmar-plantar quality of life index (PPQLI),^8^ which consists of 15-items addressing hand functionality, pain, psychological and social aspects (PPQLI-h), and 14-items assessing foot pain and functionality (PPQLI-f). It was designed in collaboration with orthopedic surgeons specialized in hands and feet to measure the effect of skin function, as opposed to joint, muscle, or ligament function.^8^ The PPQLI utilizes a Likert scale for scoring each item, with responses ranging from 0 (no impact) to 5 (severe impact), producing a score from 0 to 75 for PPQLI-h, and 0 to 70 for PPQLI-f. Higher scores indicate a greater level of impairment, reflecting a more significant impact on QoL.

PPQLI has not undergone psychometric validation, nor has it been adapted into languages other than English. Moreover, because the dermatology life quality index (DLQI) does not fully capture the functional impact of palm and sole involvement, a culturally adapted Brazilian version of the PPQLI is warranted.

We conducted a methodological study aimed at translating, culturally adapting, and validating the PPQLI for Brazilian Portuguese, following the guidelines of Beaton et al.^9^ The project was approved by the Research Ethics Committee of Universidade Estadual do Oeste do Paraná (UNIOESTE) (nº544.748), and informed consent was obtained from all participants.

After obtaining authorization from the developer, the PPQLI was translated into Portuguese by two dermatologists and one non-specialist, all fluent in English. The translations were synthesized into a consensus version, which was then presented to ten patients with PP to assess the clarity of wording and cultural appropriateness. The final version was back-translated into English by a professional translator and compared with the original tool to ensure semantic equivalence. The adapted Brazilian Portuguese version (PPQLI-BRA) is available at https://doi.org/10.17632/c4mj4vfrp3.1.

For content validation, six dermatologists with experience in psoriasis rated the relevance of each item using a Likert scale from 1 (not relevant) to 5 (highly relevant).

The PPQLI-BRA was applied to 74 patients aged ≥ 18-years, with dermatologist-confirmed PP, recruited by convenience sampling from the dermatology outpatient clinics of UNIOESTE, Cascavel, PR, Brazil, and the Hospital de Dermatologia Sanitária do Paraná (HDSPR), Piraquara, PR, Brazil, between March 2024 and November 2025. Clinical and epidemiological data, along with the DLQI-BRA, were also collected for concurrent validation. All questionnaires were self-administered.

A subgroup of 11 participants was reassessed within 21-days to estimate temporal reliability, while 16 patients under therapy were re-evaluated after 28 days to determine sensitivity to clinical change.

Internal consistency was assessed using McDonald’s ω coefficient. Temporal stability was assessed using the Intraclass Correlation Coefficient (ICC). Responsiveness was evaluated using the Wilcoxon signed-rank test. Statistical significance was set as p < 0.05. Sample size was *a priori* defined as five patients per item, and confirmed by the Kaiser-Meyer-Olkin (KMO) coefficient > 0.7, for Exploratory Factor Analysis (EFA).

Demographic, QoL, and clinical data are presented in Table 1. Item-specific scores are shown in Fig. 1. The item f28 (“use of a cane or walker to move”) has shown ground effect. During the application, PPQLI-BRA was completed in less than ten minutes.

**Table 1:** Demographic, clinical, and quality of life data of the 74 patients with palmoplantar psoriasis.

Variables		Values
Gender, *n* (%)	Female	51 (69%)
	Male	23 (31%)
Age (years), mean (SD)		57 (11)
Disease length, *n* (%)	<1 year	5 (7%)
	1‒5 years	22 (30%)
	5‒10 years	18 (24%)
	>10 years	29 (39%)
Psoriasis phenotypes, *n* (%)	Plaques	38 (51%)
	Nail	26 (35%)
	Arthritis	14 (19%)
Palmoplantar phenotypes, *n* (%)	Non-pustular	63 (85%)
	Pustular	7 (10%)
	Mixed/Overlap	4 (5%)
Topography, *n* (%)	Hands	8 (11%)
	Feet	22 (29%)
	Both	45 (60%)
Current treatments, *n* (%)	Topical corticosteroids	43 (58%)
	Topical calcipotriol	18 (24%)
	No treatment	13 (18%)
	Acitretin	10 (14%)
	Immunobiologics	12 (16%)
	Metothrexate	9 (12%)
	JAK inhibitors (oral)	1 (1%)
PPQLI-BRA-h, median (Q1‒Q3)		30 (23‒41)
PPQLI-BRA-f, median (Q1‒Q3)		29 (18‒43)
DLQI-BRA, median (Q1‒Q3)		7 (3‒14)

SD, Standard Deviation; Q1‒Q3, First and third quartiles.

**Fig. 1:**
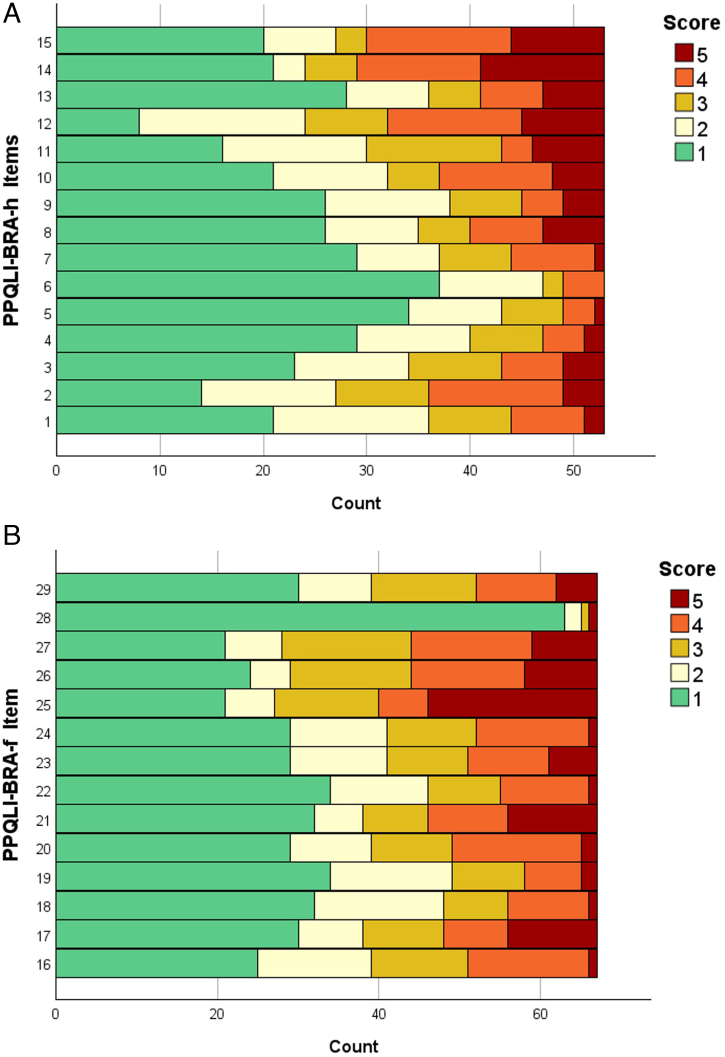
Distribution of item scores of patients: (A) PPQLI-BRA-h (*n* = 53), and (B) Distribution of item scores of patients: PPQLI-BRA-f (*n* = 67).

Content validation was confirmed with a mean expert rating > 4 for all items except for item f28.

The internal consistencies were 0.95 for PPQLI-BRA-h and PPQLI-BRA-f, and 0.91 for the DLQI. The correlations (Spearman's rho) between the DLQI scores were 0.66 and 0.56 (p < 0.01) for PPQLI-BRA-h and PPQLI-BRA-f. The inter-item correlations of the PPQLI-BRA scores, along with the correlations between each item and the total score (Fig. 2), indicate lower coefficients related to the items f25 (“avoided pedicure or feet exposition”) and f28.

**Fig. 2:**
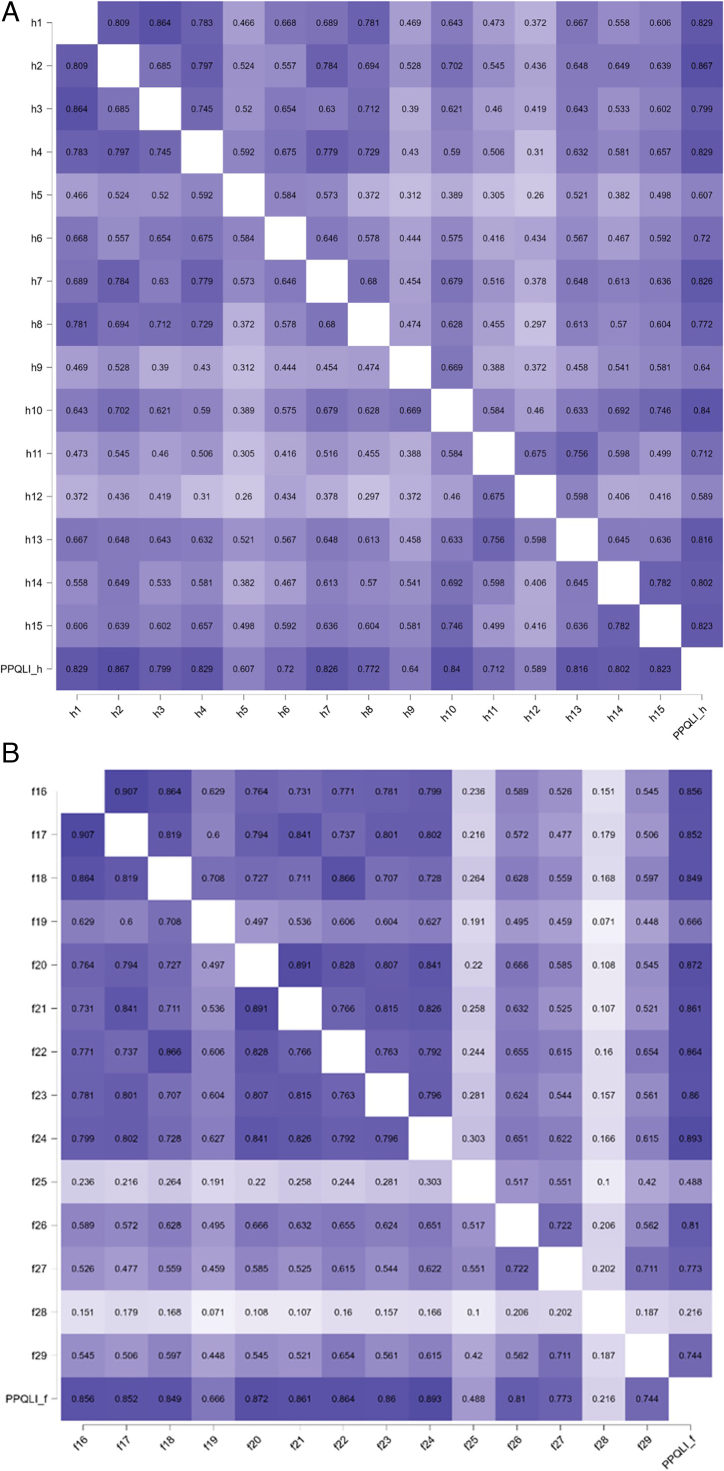
Heatmap of correlations (Spearman’s rho) between the items and the total score: (A) PPQLI-BRA-h and (B) PPQLI-BRA-f.

EFA using the principal axis factoring method based on polychoric correlation and varimax rotation, indicated that 66% (PPQLI-BRA-h) and 65% (PPQLI-BRA-f) of the construct variances were explained by the unidimensional factor. The KMO coefficient for the matrix was 0.89 for both subscales, and the sphericity test (Bartlett) was p < 0.001, indicating sample adequacy for analysis. All items but f28 showed a factor loading > 0.4. Parallel analysis supported a unidimensional structure for both subscales.

The network analysis performed using the EBICglasso method (Fig. 3) demonstrated an adequate separation between PPQLI-BRA-h and DLQI items. Item h12 (“burning/itching”) was correlated with D1 (“itching/pain”), while h9 (“social activities”) showed associations with D2 (“embarrassing”) and D3 (“going shopping/gardening”). Additionally, f25 correlated with D2 and f26 (“interfere with working”) with D7 (“working/studying”). Item f28, however, did not cluster with the PPQLI-BRA-f items and showed no correlations with other variables.

**Fig. 3:**
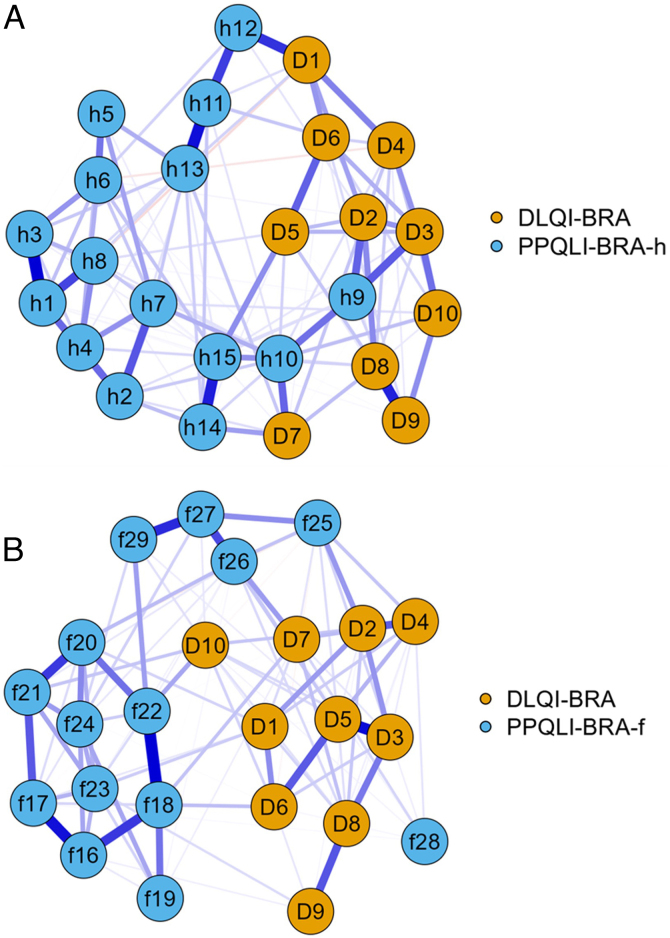
Network diagram between DLQI-BRA and PPQLI-BRA-h items (A) and PPQLI-BRA-f items (B).

In the temporal stability assessment, the ICC resulted in 0.91 for PPQLI-BRA-h and 0.96 for PPQLI-BRA-f (p < 0.01). After treatment, the assessment of sensitivity to change resulted in a mean (SD) score which varied from 47 (14) to 33 (17) in PPQLI-BRA-h, and from 43 (12) to 31 (11) for PPQLI-BRA-f (p < 0.01).

The application of the PPQLI aligns with current recommendations, which emphasize that, in addition to objective measures, patients´ self-assessment of disease impact on QoL should be considered, as this perspective can greatly influence medical decisions and healthcare systems.^10^

PPQLI-BRA enables clinicians to quantify functional impairment in palmoplantar psoriasis, guiding individualized therapeutic decisions, and its validation provides an essential tool for evaluating the QoL in patients with PP, which is underrepresented in clinical trials. The strong internal consistency and reproducibility observed confirm its psychometric robustness, comparable to other instruments, such as DLQI, which is the standard QoL measure used in psoriasis research and clinical practice in Brazil.^11^ Unlike general QoL tools, PPQLI captures unique functional limitations related to manual dexterity and ambulation, which are often underestimated in global measures.^12^

This study has limitations related to sampling patients from public referral centers and the underrepresentation of pustular phenotypes, which can hinder generalizability, although it didn’t compromise the psychometric findings. The comparison of the performance of PPQLI-BRA with other psoriasis-specific QoL instruments (e.g., PDI or PSORIQoL) was not feasible because none of these instruments has yet been culturally adapted and psychometrically validated for Brazilian Portuguese, which limits their applicability in Brazilian populations.

Future studies should explore correlations between PPQLI scores and clinical and affective variables, since PP may represent a chronic burden that predisposes to psychiatric comorbidities.

In conclusion, the Brazilian Portuguese version of the PPQLI proved to be a valid and reliable instrument for both clinical practice and research in patients with PP.

## Authors’ contributions

Paula Hitomi Sakiyama: Conception and study design; data collection, analysis, and interpretation; statistical analysis; manuscript drafting; critical revision of the manuscript for important intellectual content; critical review of the literature; approval of the final version of the manuscript.

Caio Cesar Silva de Castro: Conception and study design; data collection, analysis, and interpretation; critical revision of the manuscript for important intellectual content; critical review of the literature; approval of the final version of the manuscript.

Helena Zenedin Marchioro: Conception and study design; data collection, analysis, and interpretation; critical revision of the manuscript for important intellectual content; critical review of the literature; approval of the final version of the manuscript.

Hélio Amante Miot: Conception and study design; data collection, analysis, and interpretation; statistical analysis; manuscript drafting; critical revision of the manuscript for important intellectual content; critical review of the literature; approval of the final version of the manuscript.

## Ethical statement

Approved by the Institutional Research Ethics Committee (nº6.544.748) – UNIOESTE.

## Financial support

This work was supported by the Fundo de Apoio à Dermatologia (FUNADERM) – Sociedade Brasileira de Dermatologia.

## Research data availability

The entire dataset supporting the results of this study was published in this article.

## Conflicts of interest

None declared.
